# Polymorphisms of the matrix metalloproteinase genes are associated with essential hypertension in a Caucasian population of Central Russia

**DOI:** 10.1038/s41598-021-84645-4

**Published:** 2021-03-04

**Authors:** Maria Moskalenko, Irina Ponomarenko, Evgeny Reshetnikov, Volodymyr Dvornyk, Mikhail Churnosov

**Affiliations:** 1 Department of Medical Biological Disciplines, Belgorod State University, 308015 Belgorod, Russia; 2 Department of Life Sciences, College of Science and General Studies, Alfaisal University, Riyadh, 11533 Saudi Arabia

**Keywords:** Genetic association study, Genetic interaction

## Abstract

This study aimed to determine possible association of eight polymorphisms of seven *MMP* genes with essential hypertension (EH) in a Caucasian population of Central Russia. Eight SNPs of the *MMP1*, *MMP2*, *MMP3*, *MMP7*, *MMP8*, *MMP9*, and *MMP12* genes and their gene–gene (epistatic) interactions were analyzed for association with EH in a cohort of 939 patients and 466 controls using logistic regression and assuming additive, recessive, and dominant genetic models. The functional significance of the polymorphisms associated with EH and 114 variants linked to them (r^2^ ≥ 0.8) was analyzed in silico. Allele G of rs11568818 *MMP7* was associated with EH according to all three genetic models (OR = 0.58–0.70, p_perm_ = 0.01–0.03). The above eight SNPs were associated with the disorder within 12 most significant epistatic models (OR = 1.49–1.93, p_perm_ < 0.02). Loci rs1320632 *MMP8* and rs11568818 *MMP7* contributed to the largest number of the models (12 and 10, respectively). The EH-associated loci and 114 SNPs linked to them had non-synonymous, regulatory, and eQTL significance for 15 genes, which contributed to the pathways related to metalloendopeptidase activity, collagen degradation, and extracellular matrix disassembly. In summary, eight studied SNPs of *MMPs* genes were associated with EH in the Caucasian population of Central Russia.

## Introduction

Cardiovascular diseases are a global problem of modern healthcare and the second most common cause of total mortality^1,2^. Among cardiovascular diseases, essential hypertension (EH) is of tremendous clinical importance in terms of health, working capacity, and life expectancy^3–5^. According to some estimates, the number of patients with EH will reach 1.56 billion by 2025^6^. More than 9.4 million death cases resulting from EH complications, such as stroke, myocardial infarction, renal failure, etc., are recorded worldwide annually^7–9^.

The development of EH is determined by complex interaction mechanisms of genetic and environmental factors^5,10–12^. The contribution of hereditary factors to EH is estimated from 25 to 75% in different populations according to family and twin studies^13,14^. Among the possible candidate genes for EH are matrix metalloproteinases (*MMP*). This is a group of enzymes with a wide range of biological functions that are responsible for the hydrolysis of the extracellular matrix (ECM) components^15–18^. The imbalance between synthesis and degradation of ECM caused by the change in MMP gene expression can lead to a decrease in the ability of the vascular wall to remodel and to the development of cardiovascular diseases^19,20^. It was found that single nucleotide polymorphisms (SNP) of the *MMP* genes are associated with EH and its complications in different populations: Australian (rs3025058 *MMP3*), Polish (rs3025058 *MMP3*), American (rs652438 *MMP12*), Swedish (rs11568818 *MMP7*), Brazilian (rs243865 *MMP2*), Serbian (rs11225395 and rs1320632 *MMP8*, rs1799750 *MMP1*) and others^16,19,21–26^. However, no associations of the *MMP* polymorphisms with EH and its complications were found in Chinese (rs3025058 *MMP3*, rs17577 *MMP9*), Indian (rs11568818 *MMP7*), and Mexican (rs1799750 *MMP1*) populations^27–29^. The observed inconsistencies prompt for further studies of the role of the *MMP* polymorphisms in the development of EH.

In this study, we analyzed the associations of matrix metalloproteinases and their gene–gene interactions with EH in a Caucasian population of Central Russia. We also examined the biological mechanisms of their phenotypic effects and SNPs linked to them (nonsynonymous substitutions, regulatory and *cis*-eQTL influences, pathways).

## Results

### Study participants characteristics

The patients with EH had higher rates of BMI, total cholesterol, triglycerides, low-density lipoprotein cholesterol, low high-density lipoprotein cholesterol, and a high incidence of smokers (p < 0.001) (Table 1). These factors were applied as covariates in the association analyses.

**Table 1:** Phenotypic characteristics of the study participants.

Parameters	EH, mean ± SD, % (n)	Controls, mean ± SD, % (n)	p
N	939	466	–
Gender (male/female)	60.06 /39.94 (564/375)	55.15/44.85 (257/209)	0.09
Age (years)	58.08 ± 8.91	57.82 ± 9.52	0.77
BMI (kg/m^2^)	30.78 ± 5.08	24.94 ± 3.14	< 0.001
SBP (mmHg)	182.48 ± 28.26	122.58 ± 11.49	< 0.001
DBP (mmHg)	105.84 ± 13.47	77.65 ± 6.93	< 0.001
TC (mM)	5.71 ± 1.29	5.26 ± 1.04	< 0.001
HDL-C (mM)	1.34 ± 0.42	1.52 ± 0.42	< 0.001
LDL-C (mM)	3.78 ± 1.11	3.22 ± 0.74	< 0.001
TG (mM)	1.92 ± 1.03	1.22 ± 0.71	< 0.001
Blood glucose (mM)	5.92 ± 1.68	4.88 ± 0.95	< 0.001
Smoking	38.33 (353)	19.76 (84)	< 0.001
Alcohol abuse	5.79 (53)	3.12 (13)	0.051

Clinical characteristics of age, BMI, SBP, DBP, HDL-C, LDL-C, TG and TC are given as means ± SD and other values as number of individuals.

*BMI*, body mass index, *SBP*, systolic blood pressure, *DBP*, diastolic blood pressure; *TC*, total cholesterol, *HDL-C*, high-density lipoprotein cholesterol, *TG*, triglycerides, *LDL-C*, low-density lipoprotein cholesterol.

### Analysis of SNP association

The data about the studied SNPs is given in Table 2. All loci had MAF > 5% and showed no departure from the HWE (p > 0.05).

**Table 2:** The allele and genotype frequencies (%) of the studied SNPs in the EH and control groups.

SNP	Genotypes, indicators	EH, % (n)	Controls, % (n)
rs11568818 *MMP7*	AA	36.38 (338)	31.82 (147)
	AG	48.65 (452)	49.13 (227)
	GG	14.97 (139)	19.05 (88)
	Minor allele G	39.29 (730)	43.61 (403)
	H_o_/H_e_ (p_HWE)_	0.49/0.48 (p > 0.05)	0.49/0.49 (p > 0.05)
rs1320632 *MMP8*	AA	84.67 (784)	81.48 (374)
	AG	14.47 (134)	17.65 (81)
	GG	0.86 (8)	0.87 (4)
	Minor allele G	8.10 (150)	9.69 (89)
	H_o_/H_e_ (p_HWE)_	0.14/0.15 (p > 0.05)	0.18/0.17 (p > 0.05)
rs11225395 *MMP8*	CC	31.09 (291)	25.97 (120)
	CT	48.50 (454)	48.27 (223)
	TT	20.41 (191)	25.76 (119)
	Minor allele T	44.66 (836)	49.89 (461)
	H_o_/H_e_ (p_HWE)_	0.48/0.49 (p > 0.05)	0.48/0.50 (p > 0.05)
rs1799750 *MMP1*	1G/1G	28.01 (263)	28.73 (133)
	1G/2G	49.20 (462)	47.30 (219)
	2G/2G	22.79 (214)	23.97 (111)
	Minor allele 2G	890 (47.39)	47.62 (441)
	H_o_/H_e_ (p_HWE)_	0.49/0.50 (p > 0.05)	0.47/0.50 (p > 0.05)
rs3025058 *MMP3*	6A/6A	30.63 (287)	26.73 (127)
	5A/6A	48.77 (457)	50.53 (240)
	5A/5A	20.60 (193)	22.74 (108)
	Minor allele 5A	44.98 (843)	48.00 (456)
	H_o_/H_e_ (p_HWE)_	0.48/0.50 (p > 0.05)	0.51/0.50 (p > 0.05)
rs652438 *MMP12*	AA	86.97 (814)	90.20 (414)
	AG	12.39 (116)	9.37 (43)
	GG	0.64 (6)	0.43 (2)
	Minor allele G	128 (6.84)	5.12 (47)
	H_o_/H_e_ (p_HWE)_	0.12/0.13 (p > 0.05)	0.09/0.10 (p > 0.05)
rs243865 *MMP2*	CC	58.15 (546)	59.57 (277)
	CT	35.14 (330)	35.05 (163)
	TT	6.71 (63)	5.38 (25)
	Minor allele T	24.28 (456)	25.66 (213)
	H_o_/H_e_ (p_HWE)_	0.35/0.37 (p > 0.05)	0.39/0.38 (p > 0.05)
rs17577 *MMP9*	GG	66.95 (622)	61.00 (280)
	AG	29.82 (277)	34.64 (159)
	AA	3.23 (30)	4.36 (20)
	Minor allele A	18.14 (337)	21.68 (199)
	H_o_/H_e_ (p_HWE)_	0.30/0.30 (p > 0.05)	0.35/0.34 (p > 0.05)

The number in the brackets represents the number of participants with the respective genotype (or allele).

The allele G of the rs11568818 *MMP7* polymorphism was associated with EH according to the additive (OR = 0.70, 95% CI 0.54–0.89, p = 0.001, p_perm_ = 0.01, power 99.03%), dominant (OR = 0.66, 95% CI 0.46–0.97, p = 0.02, p_perm_ = 0.03, power 94.50%) and recessive (OR = 0.58, 95% CI 0.37–0.91, p = 0.01, p_perm_ = 0.02, power 91.54%) models (Table 3).

**Table 3:** Association of the *MMPs* genotypes with EH.

SNPs	Model	Genotype	OR (95% CI)	p
rs11568818 *MMP7*	Dominant	AG/GG vs AA	0.66 (0.46–0.97)	0.02
	Recessive	GG vs AG/AA	0.58 (0.37–0.91)	0.01
	Additive	AG vs GG vs AA	0.70 (0.54–0.89)	0.001
rs1320632 *MMP8*	Dominant	GA/GG vs AA	0.66 (0.42–1.05)	0.07
	Recessive	GG vs GA/AA	0.84 (0.09–8.27)	0.99
	Additive	GA vs GG vs AA	0.69 (0.45–1.06)	0.08
rs11225395 *MMP8*	Dominant	CT/TT vs CC	0.74 (0.50–1.10)	0.13
	Recessive	TT vs CT/CC	0.69 (0.46–1.04)	0.15
	Additive	CT vs TT vs CC CT vs TT vs CC	0.78 (0.61–1.00)	0.07
rs1799750 *MMP1*	Dominant	1G2G/2G2G vs 1G1G	1.07 (0.73–1.56)	0.71
	Recessive	2G2G vs 1G2G /1G1G	1.02 (0.68–1.53)	0.86
	Additive	1G2G vs 2G2G vs 1G1G	1.03 (0.81–1.31)	0.70
rs3025058 *MMP3*	Dominant	5A6A /5A5A vs 6A6A	1.12 (0.77–1.62)	0.61
	Recessive	5A5A vs 5A6A/6A6A	0.87 (0.57–1.34)	0.69
	Additive	5A6A vs 6A6A vs 5A5A	1.01 (0.79–1.28)	0.75
rs652438 *MMP12*	Dominant	GA/GG vs AA	1.46 (0.81–2.61)	0.26
	Recessive	GG vs GA/AA	1.34 (0.10–1.74)	0.86
	Additive	GA vs GG vs AA	1.41 (0.81–2.44)	0.19
rs243865 *MMP2*	Dominant	CT/TT vs CC	1.04 (0.73–1.48)	0.86
	Recessive	TT vs CT/CC	1.55 (0.71–3.39)	0.26
	Additive	CT vs TT vs CC	1.09 (0.82–1.46)	0.85
rs17577 *MMP9*	Dominant	AG/AA vs GG	0.94 (0.66–1.35)	0.85
	Recessive	AA vs AG/GG	0.91 (0.36–2.32)	0.74
	Additive	AG vs GG vs AA	0.95 (0.69–1.29)	0.68

*OR* odds ratio, *95% CI* 95% confidence interval, *p* significance level.

### SNP × SNP interactions

In total 12 most significant two-, three, and four-locus models of gene–gene interactions associated with EH were determined (p_perm_ ≤ 0.02, cross-validation consistency CVC = 10/10, testing balanced accuracy 49.2–55.3%) (Tables 4 and 5). These models included eight SNPs. Loci rs1320632 *MMP8* and rs11568818 *MMP7* were involved in the largest number of models (12 and 10, respectively). Loci rs652438 *MMP12*, rs11225395 *MMP8*, rs1320632 *MMP8*, and rs11568818 *MMP7* contributed to the most significant epistatic models (p_perm_ = 0.002). The following combinations of genotypes had most significant associations with EH: rs11225395 CT × rs1320632 AG × rs11568818 CC (beta = − 0.827, p = 0.002), rs652438 AA × rs11225395 CT × rs1320632 AG × rs11568818 CT (beta = − 0.847, p = 0.003), rs17577 AG × rs1320632 AG × rs11568818 CT × rs1799750 1G/1G (beta = − 1.726, p = 0.002) (Supplementary Table 1).

**Table 4:** Gene–gene interactions significantly associated with the risk of EH according to MB-MDR.

N	SNP x SNP interaction models	NH	*beta*H	WH	NL	*beta*L	WL	Pperm
**Two-order interaction models**								
1	rs11225395 *MMP8* × rs1320632 *MMP8*	1	0.242	2.74	2	− 0.51	10.54	0.008
2	rs1320632 *MMP8* × rs11568818 *MMP7*	0	NA	NA	3	− 0.55	10.77	0.010
**Three-order interaction models**								
3	rs1320632 *MMP8* × rs11568818 *MMP7* × rs3025058 *MMP3*	1	0.922	3.36	5	− 0.907	23.88	0.002
4	rs11225395 *MMP8* × rs1320632 *MMP8* × rs11568818 *MMP7*	1	0.497	4.09	3	− 0.715	16.88	0.004
5	rs17577 *MMP9* × rs1320632 *MMP8* × rs11568818 *MMP7*	0	NA	NA	2	− 0.927	16.22	0.004
6	rs1320632 *MMP8* × rs11568818 *MMP7* × rs1799750 *MMP1*	0	NA	NA	3	− 0.816	15.66	0.006
7	rs11225395 *MMP8* × rs1320632 *MMP8* × rs3025058 *MMP3*	2	0.460	9.72	4	− 0.634	16.13	0.012
**Four-order interaction models**								
8	rs652438 *MMP12* × rs11225395 *MMP8* × rs1320632 *MMP8* rs11568818 *MMP7*	1	0.363	3.33	4	− 0.862	23.48	0.002
9	rs652438 *MMP12* × rs1320632 *MMP8* × rs11568818 *MMP7* × rs3025058 *MMP3*	0	NA	NA	5	− 0.918	22.06	0.004
10	rs1320632 *MMP8* × rs11568818 *MMP7* × rs3025058 *MMP3* × rs243865 *MMP2*	2	0.794	7.05	5	− 1.311	26.38	0.006
11	rs11225395 *MMP8* × rs1320632 *MMP8* × rs11568818 *MMP7* × rs243865 *MMP2*	2	1.089	7.96	4	− 1.090	23.26	0.006
12	rs17577 *MMP9* × rs1320632 *MMP8* × rs11568818 *MMP7* × rs1799750 *MMP1*	0	NA	NA	4	− 1.261	21.38	0.018

*NH*, number of significant High risk genotypes in the interaction, *beta H*, regression coefficient for High risk exposition in the step 2 analysis, *NA* – not available, *WH*, Wald statistic for High risk category, *NL*, number of significant Low risk genotypes in the interaction, *beta L*, regression coefficient for Low risk exposition in the step 2 analysis, *WL*, Wald statistic for Low risk category, *Pperm*, Permutation P-value for the interaction model.

**Table 5:** Cross-validation statistics for best models of the gene–gene interactions in EH.

N	SNP × SNP interaction models	OR (95% CI)	Testing balanced accuracy	Cross-validation consistency	Pperm
**Two-order interaction models**					
1	rs11225395 *MMP8* × rs1320632 *MMP8*	1.57 (1.23–1.99)	0.543	10/10	0.008
2	rs1320632 *MMP8* × rs11568818 *MMP7*	1.49 (1.16–1.92)	0.537	10/10	0.01
**Three-order interaction models**					
3	rs1320632 *MMP8* × rs11568818 *MMP7* × rs3025058 *MMP3*	1.54 (1.22–1.94)	0.511	10/10	0.002
4	rs11225395 *MMP8* × rs1320632 *MMP8* × rs11568818 *MMP7*	1.70 (1.35–2.15)	0.540	10/10	0.004
5	rs17577 *MMP9* × rs1320632 *MMP8* × rs11568818 *MMP7*	1.72 (1.34–2.22)	0.531	10/10	0.004
6	rs1320632 *MMP8* × rs11568818 *MMP7* × rs1799750 *MMP1*	1.51 (1.19–1.91)	0.492	10/10	0.006
7	rs11225395 *MMP8* × rs1320632 *MMP8* × rs3025058 *MMP3*	1.73 (1.38–2.17)	0.553	10/10	0.012
**Four-order interaction models**					
8	rs652438 *MMP12* × rs11225395 *MMP8* × rs1320632 *MMP8* rs11568818 *MMP7*	1.75 (1.40–2.20)	0.537	10/10	0.002
9	rs652438 *MMP12* × rs1320632 *MMP8* × rs11568818 *MMP7* × rs3025058 *MMP3*	1.71 (1.37–2.14)	0.529	10/10	0.004
10	rs1320632 *MMP8* × rs11568818 *MMP7* × rs3025058 *MMP3* × rs243865 *MMP2*	1.78 (1.40–2.23)	0.503	10/10	0.006
11	rs11225395 *MMP8* × rs1320632 *MMP8* × rs11568818 *MMP7* × rs243865 *MMP2*	1.93 (1.54–2.41)	0.531	10/10	0.006
12	rs17577 *MMP9* × rs1320632 *MMP8* × rs11568818 *MMP7* × rs1799750 *MMP1*	1.73 (1.38–2.16)	0.492	10/10	0.018

Models are obtained using the multifactor dimensionality reduction method, version 3.0.2

*CI*, confidence interval, *OR*, odds ratio, *Pperm*, permutation P-value for the interaction model.

**Figure 1:**
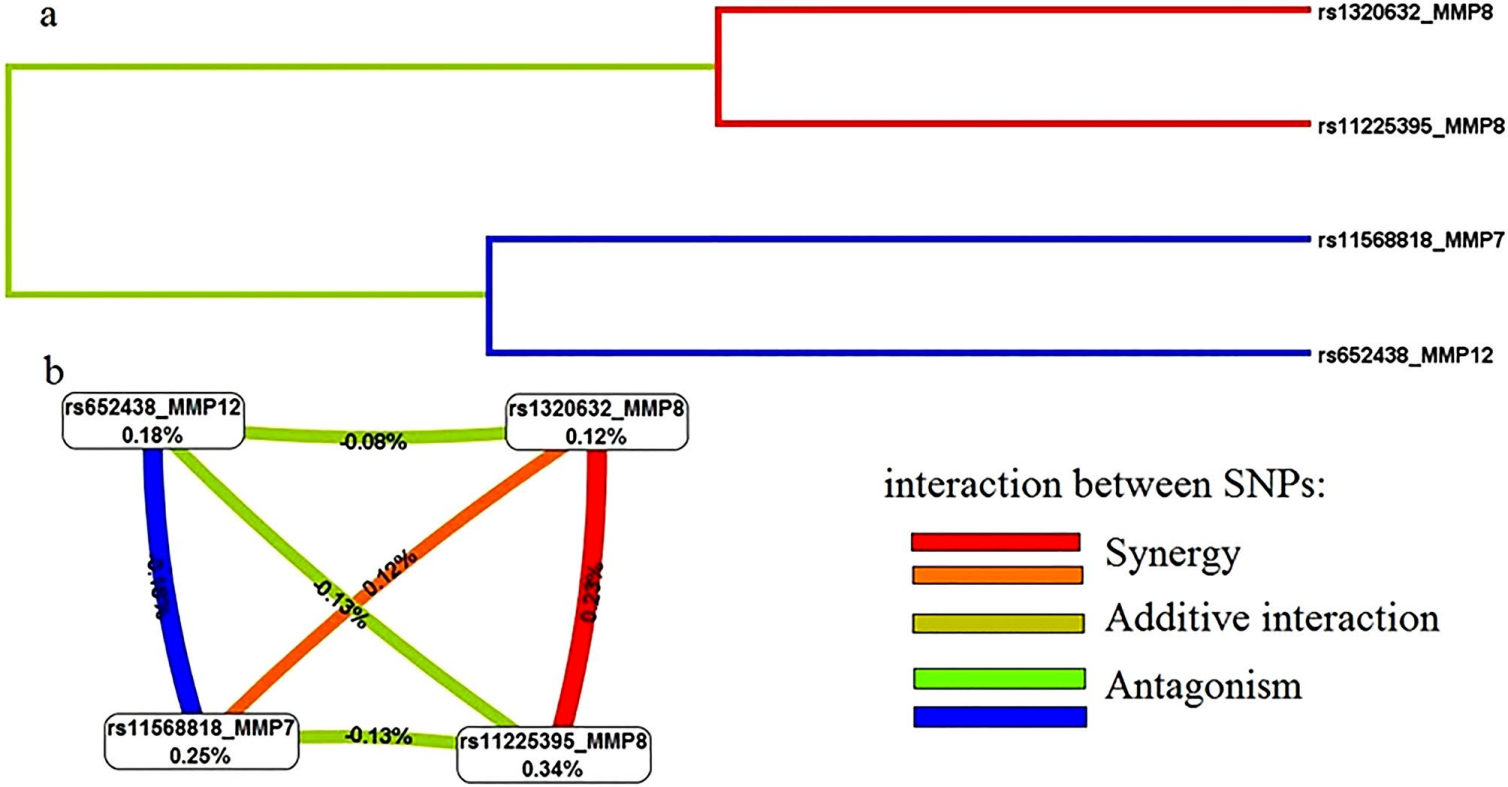
High-order gene–gene interaction analysis for SNPs of matrix metalloproteinases in EH (data obtained by Multifactor Dimensionality Reduction, version 3.0.2): (**a**) Interaction dendrogram; (**b**) Interaction entropy graph.

The graph of the most significant epistatic models of the four SNPs (Fig. 1) suggests these interactions are concerted. Pronounced synergism was observed between the polymorphisms of the *MMP8* gene, while an antagonistic interaction was suggested between rs652438 *MMP12* and rs11568818 *MMP7*. The graph of interactions (Fig. 1b) shows that rs11225395 *MMP8* and rs11568818 *MMP7* eliminated 0.34 and 0.25% of class entropy, respectively, thereby having the largest univariate effects.

### Functional SNP

#### Non-synonymous SNPs

Among the eight SNPs studied, two loci (rs17577 *MMP9* and rs652438 *MMP12*) cause the replacement of amino acids in the encoded polypeptide and a decrease in its activity (Supplementary Table 2). Also, three non-synonymous variants (rs679620 *MMP3*, rs1940475 and rs3765620 *MMP8*) were determined among the polymorphisms linked to the studied SNPs (Supplementary Table 2).

#### Regulatory effects

The data on the regulatory effects of the EH-associated loci are presented in Supplementary Table 3. According to the HaploReg database (v4.1), two SNPs were located in evolutionarily conserved regions, two polymorphisms—in the promoter histone marks region, and eight SNPs—in the enhancer histone marks region in various tissues. Seven SNPs were in the hypersensitivity region to DNAse-1, three SNPs—in the protein-bound region, five SNPs—in the motifs changed region. According to the SNPinfo Web Server database, polymorphisms rs1320632 *MMP8* and rs11225395 *MMP8* possessed the most significant regulatory potential (0.53 and 0.20, respectively). Four SNPs were located in the regions of the transcription factor binding site (TFBS), one was in the microRNA binding region, and two were in the exonic splicing enhancer and exonic splicing silencer. SNP rs11568818 was located in the DNA regulatory motifs region: the allele G increased affinity to transcription factors Foxa (ΔLOD scores = − 5.1), PLZF (ΔLOD scores = − 1.5), Pou5f1 (ΔLOD scores = − 3.2) and reduced affinity to GR transcription factor (ΔLOD scores = 0.8).

In addition to the eight EH-associated SNPs, regulatory significance was estimated for 114 polymorphisms linked to them (Supplementary Table 4). Eight SNPs (including five non-synonymous and three synonymous substitutions) were located in exons of the studied genes, three were located in 3′-UTR and one in 5′-UTR, 86 were in introns, and 44 were in intergenic regions. Nine loci were located in evolutionarily conserved regions.

The in silico analysis of SNPs linked to the EH-associated loci suggested several polymorphisms with pronounced regulatory effects (Supplementary Table 4). For example, rs243862 (linked to rs243865 *MMP2*) is located in the promoter histone marks region in 21 tissues and enhancer histone marks in two tissues, the hypersensitivity region to DNAse-1 in 19 tissues, region 14 motifs changed.

#### Expression QTLs

 According to the GTExportal database, four EH-associated SNPs had the cis-eQTL significance (p < 8 × 10^–5^, pFDR ≤ 0.05) and might affect the expression of five genes (*MMP7*, *MMP27*, *RP11-817J15.3*, *SNX21*, *SLC12A5*) in several tissues and organs (Supplementary Table 5). Six EH-associated loci were in strong LD with the SNPs affecting the expression (p < 8.5 × 10^–5^, FDR ≤ 0.05) of 11 genes in more than 20 tissues and organs, including those pathogenetically significant for the development of EH (whole blood, tibial artery, left ventricle of heart, etc.) (Supplementary Table 6).

*Pathway analyses.* The in silico analysis of the functional significance was conducted for the 7 EH-associated genes (*MMP7*, *MMP8*, *MMP1*, *MMP2*, *MMP3*, *MMP9*, *MMP12*) and for genes whose expression is affected by the EH-associated SNPs according to the eQTL analysis (Supplementary Tables 5 and 6).

Of the 15 genes considered, the Gene Ontology databases have the relevant information about 12 genes; the information about three genes (*RP11-817J15*, *WTAPP1,* and *RPL13P2*) was not available. We found evidence of enrichment for pathways involved in the metalloendopeptidase activity (P_FDR_ = 1.37E−16), collagen catabolic process (P_FDR_ = 4.68E−15), collagen degradation (P_FDR_ = 8.92E−15), extracellular matrix disassembly (P_FDR_ = 4.79E−14), activation of matrix metalloproteinases (P_FDR_ = 2.28E−13), and 10 other pathways (FDR ≤ 0.05) (Supplementary Table 7).

**Figure 2:**
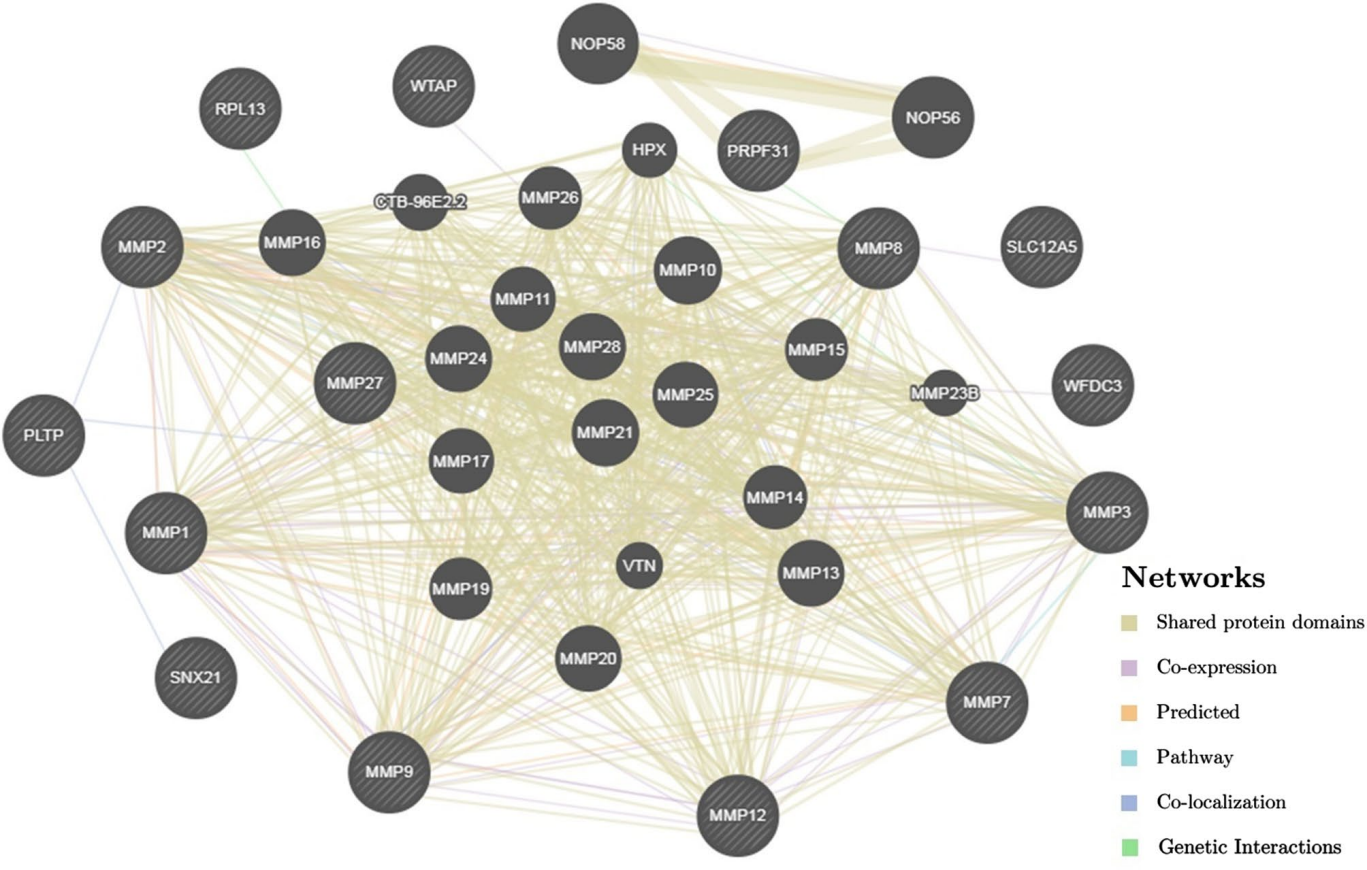
The interaction network of the candidate genes for EH obtained using the GeneMANIA online service, http://www.genemania.org/ (the genes associated with EH according to the present study are cross-shaded).

The network of the intergenic interactions between the 15 EH-associated genes and other 20 genes inferred by GeneMANIA is given in Fig. 2. These interactions are realized through common protein domains (47.03%), co-expression (35.73%), pathogenetic pathways (6.11%), co-localization (2.63%), and genetic interactions (0.53%). The EH-associated genes may interact either directly or via other genes (e.g., *NOP56*, *NOP58*, *MMP10*, *MMP21*, *MMP28*, etc*.*). Among the 35 candidate genes for EH, the most significant interactions were determined for *NOP56* and *NOP58* (common protein domains, weight = 0.65), *PRPF31* и *NOP56* (common protein domains, weight = 0.41), *PRPF31* и *NOP58* (common protein domains, weight = 0.41), *MMP7* и *MMP3* (pathways, weight = 0.10).

## Discussion

The present study determined significant associations of eight loci of matrix metalloproteinase genes with EH in a Caucasian population of Central Russia.

Allele G of locus rs11568818 *MMP7* was associated with EH according to the dominant, additive and recessive models (OR = 0.58–0.70) and was involved in 10 of 12 two-, three-, and four-locus models of gene–gene interactions associated with EH. Marker rs11568818 was characterized by a significant regulatory effect: it was located in the region hypersensitive to DNAse-1 in 15 tissues, in the region of modified histones (H3K4me1 and H3K4me3) that marked promoter and enhancer in 11 different organs and tissues, and affected the *MMP7* gene expression. The locus was located in the region of DNA that binds to the TATA-binding protein (TBP), c-FOS, c-Jun, and located in regulatory protein binding sites. According to the GeneCards database, TBP is responsible for proper RNA polymerase positioning on a promoter during transcription; regulatory proteins c-FOS and c-Jun interact with each other and control cell proliferation, differentiation, and transformation. The data about the possible contribution of rs11568818 to cardiovascular disease in different ethnic populations was somewhat inconsistent. For example, Jormsjö et al.^23^ showed that carriers of genotype GG rs11568818 *MMP7* had an increased risk for developing cardiovascular pathology in the Swedish population, while in populations from India, Mexico, and Turkey associations of this marker with EH and its complications were not determined^28,30,31^. The observed inconsistencies could stem from the differences in study designs (e.g., differences in the used covariates, sample sizes, gene–gene and gene-environment interactions, etc.). In addition, the differences in the results might be associated with ethnicity-specific pathogenetic features of the emergence and course of EH^32–37^ or/and ethnicity-related differences in the genetic structure of the populations^38,39^.

According to the GeneCards database (http://www.genecards.org/), the *MMP7* gene belongs to the gene cluster on chromosome 11 and encodes the enzyme of the same name, which is characterized by the absence of a conserved C-terminal hemopexin domain. Matrix metalloproteinase 7 is responsible for proteolytic cleavage of elastin, type I, III, IV, V gelatins, fibronectin, casein, proteoglycans and is involved in the regeneration processes after damage, remodeling of the extracellular matrix, and also modulates cell migration, proliferation, and apoptosis^15^.

We did not determine monolocus effects for rs1320632 of the *MMP8* gene; however, this marker is involved in all 12 identified gene–gene interactions models associated with EH. SNP rs1320632 has significant regulatory potential—it is located in the region of DNA regulatory motifs and modulates affinity for seven transcription factors (CAC-binding-protein, Foxc1-2, GATA-known13, GCM, MAZ, PRDM1-known1, STAT-disc6). In addition, this SNP is located in the region of hypersensitivity to DNAse-1 in 10 tissues, in the region of H3K4me1 and H3K4me3, marking enhancers and promoters in six tissues. We found that rs1320632 *MMP8* is strongly linked to 14 SNPs that have important regulatory significance, and this locus is associated with a level of the *MMP27* gene expression in four tissues. Our results are consistent with those previously reported for the Serbian population^22^. The *MMP8* gene encodes a proteolytic enzyme involved in the cleavage of the extracellular matrix in the proliferation and remodeling of tissues, embryonic development, as well as in pathological processes such as arthritis and metastasis. Selective proteolysis of the polypeptide leads to the formation of many active forms of the enzyme with different N-ends. MMP8 is involved in the degradation of type I-III collagen and is expressed by macrophages, while the production of MMP8 sharply increases with inflammation.

It should be noted that the current study is somewhat limited because: (a) only one ethnic population was analyzed. The well-known ethnic disparities in the prevalence of complex diseases warrant validation studies of the determined associations of the *MMP* genes and EH in other ethnic populations; (b) a transethnic meta-analysis of the studied *MMP* SNPs would help to clarify this issue, but is currently impossible due to the insufficient data available about *MMP* and EH; (c) the obtained results are not sufficient to construct a reliable predictive model of EH based on the eight studied SNPs using the multi-model deep learning method.

## Conclusions

Thus, in this work, we found that genetic polymorphism rs11568818 *MMP7* and gene–gene interactions of eight SNPs are associated with EH in a Caucasian population of Central Russia, and their phenotypic effects are realized through non-synonymous substitutions, regulatory and *cis*-eQTL effects, and shaed biological pathways.

## Materials and methods

### Study subjects

The study sample included 1405 people: 939 patients with essential hypertension and 466 controls. The participants were recruited through the cardiological and neurological departments of the St. Joasaph Belgorod Regional Clinical Hospital during 2013–2016. The following inclusion criteria were adopted: self-declared Russian descent, a birthplace in Central Russia^40^.

Essential hypertension was diagnosed by certified physicians in cardiology and neurology as recommended by the World Health Organization (n = 939, 100%). All study subjects had a clinical history of hypertension for more than one year. Untreated hypertensive patients had the established hypertension defined by seated systolic (SBP) and/or diastolic (DBP) blood pressure above 140 and/or 90 mm Hg, respectively, measured at least twice. All hypertensive patients had no clinical signs, symptoms, and laboratory findings suggesting secondary hypertension, and liver or/and kidney failure. The controls were recruited during regular medical examinations at the above Center. The criterion for inclusion in the control group was the level of SBP < 140 mmHg and the level of DBP < 90 mmHg, no history of metabolic syndrome, autoimmune disorders, and oncological diseases.

The level of blood pressure (BP) was determined by the auscultation method using a sphygmomanometer and according to Korotkov^41^. BP was measured throughout several days (at least twice). The patients had not consumed caffeine, exercise, and smoke for at least 30 min before the measurement procedure bean. The measurement was performed in the seated position of the patient after 5 min of rest. The blood pressure was measured on both arms: at least two measurements were taken with an interval of 1–2 min. A mean of at least two readings taken at least two times was used to assess individual blood pressure.

The study was carried out in accordance with the standards of Good Clinical Practice and the principles of the Helsinki Declaration. The study was approved by the Regional Ethics Committee of Belgorod State University. All participants signed informed consent before the enrolment in the study.

Data on anthropometric characteristics (height, weight, and body mass index), smoking and alcohol use were collected for each participant. Blood samples for determining total cholesterol (TC, mmol/l), triglycerides (TG, mmol/l), high-density lipoprotein cholesterol (HDL-C, mmol/l), and low-density lipoprotein cholesterol (LDL-C, mmol/l) were collected after 8-h fasting, the analysis was performed in the certified clinical diagnostic laboratory of the St. Joasaph Belgorod Regional Clinical Hospital. The baseline and clinical characteristics of the study population are given in Table 1. The control group was matched to the EH group for sex and age (*p* > 0.05).

### SNP selection and DNA handling

DNA was extracted from whole blood by the phenol–chloroform protocol and then checked for quality (as described earlier^42^).

Eight single nucleotide polymorphisms (SNPs) of seven matrix metalloproteinase genes (rs1799750 *MMP1*, rs243865 *MMP2*, rs3025058 *MMP3*, rs11568818 *MMP7,* rs1320632 and rs11225395 *MMP8*, rs17577 *MMP9*, and rs652438 *MMP12*) were selected for the study based on the following criteria^42,43^: (1) SNP associations with the development of EH and/or its complications according to the results of previous studies; (2) the regulatory effect of a polymorphism (regSNP); (3) the effect of the locus on gene expression (eSNP); (4) relation to non-synonymous substitutions (nsSNP); (5) MAF > 0.05. The SNPs regulatory potential and effect on the gene expression were assessed using HaploReg (v4.1) (http://archive.broadinstitute.org/mammals/haploreg/haploreg.php), SNPinfo Web Server—SNP Function Prediction (FuncPred) (https://snpinfo.niehs.nih.gov/snpinfo/snpfunc.html), and GTExportal (http://www.gtexportal.org/).

Information on the biological role of the studied polymorphisms and their associations with cardiovascular pathology is presented in Supplementary Table 8. All SNPs appeared to have significant regulatory potential, four of them (50.00%) were eSNPs, and 2 were nsSNPs (Supplementary Table 3).

### SNP genotyping

The polymorphisms were genotyped using the MALDI‐TOF mass spectrometry iPLEX platform (Agena Bioscience Inc, San Diego, CA). Genotyping of blind replicates was performed to ensure quality control. The repeatability test was performed for 5% of randomly selected samples and showed 100% reproducibility.

### Statistical analysis

Correspondence of the studied loci to the Hardy–Weinberg equilibrium (HWE) was checked by the chi-square test. The loci were analyzed for associations with EH using logistic regression and according to additive (i.e., comparison of all genotypes, e.g., TT vs TC vs CC), dominant (CC/TC vs TT, where C is a minor allele), and recessive (CC vs TC/TT, where C is a minor allele) genetic models with adjustment for covariates. The following covariates were applied as quantitative variables: BMI, total cholesterol, triglycerides, high-density and low-density lipoprotein cholesterol; and while smoking status was used as qualitative variables (yes/no) (Table 1). The adaptive permutation test^44^ was applied to adjust the results for multiple comparisons. The significance level was set at *p*_*perm*_ < 0.05. The calculations were performed using software PLINK v.2.050 (http://zzz.bwh.harvard.edu/plink/). Statistical power for each SNP was estimated using Quanto 1.2.4 (http://hydra.usc.edu/gxe, 2009).

The epistatic interactions were analyzed assuming two-, three-, and four-locus models. The MB-MDR (Model Based Multifactor Dimensionality Reduction)^45,46^ approach and respective software (v. 2.6) for the R programming environment were utilized for the computations. The significance of the gene–gene interaction models was evaluated by the permutation test^44^. For the permutation test, the following threshold *p* values (after the Bonferroni correction based on the numbers of combinations studied for eight loci) were adopted for the models of the gene–gene interactions: *p* < 1.8 × 10^–3^ (< 0.05/28) for the two-locus models, *p* < 8.9 × 10^–4^ (< 0.05/56) for the three-locus models, and *p* < 7.1 × 10^–4^ (< 0.05/70) for the four-locus models. The significance level was set at *p*_*perm*_ < 0.05.

The cross-validation of the most significant models of intergenic interactions associated with EH was conducted by MDR (Multifactor Dimensionality Reduction)^47^, as implemented in the MDR software (v.3.0.2) (http://sourceforge.net/projects/mdr). The MDR method was used to assess the nature and strength (contribution to entropy) of gene–gene interactions and visualize them in graph form^48^.

### Functional SNPs

The SNPs associated with EH and those strongly linked to them were evaluated for their functional significance (non-synonymous SNPs^49^, regulatory potential^50,51^, and eQTLs^52^). The loci in linkage disequilibrium (LD) (r^2^ ≥ 0.8) with the EH-associated ones were determined using HaploReg (v4.1) (http://archive.broadinstitute.org/mammals/haploreg/haploreg.php) and the data of the European population from the 1000 Genomes Project Phase 1^42,50,53,54^.

#### Non-synonymous SNPs

Non-synonymous SNPs and their predictive potential were analyzed using the SIFT tool (https://sift.bii.a-star.edu.sg/)^49^.

#### Regulatory effects

The candidate loci for EH were analyzed in silico for their regulatory potential using HaploReg (v4.1) (http://archive.broadinstitute.org/mammals/haploreg/haploreg.php)^50^ and SNP Function Prediction (FuncPred) (https://snpinfo.niehs.nih.gov/snpinfo/snpfunc.html)^51^.

#### Expression QTLs

The effect of the candidate SNPs for EH on gene expression in various tissues and organs was estimated using the GTExportal data (http://www.gtexportal.org/) as of 10.12.2019 (Release V8 updated on 26/08/2019) (dbGaP Accession phs000424.v8.p2)^52^. The False Discovery Rate (FDR) ≤ 0.05 was applied as statistical significance threshold^55^.

#### Pathway analyses

The genes associated with EH were analyzed for functional significance in the various metabolic pathways using the Gene Ontology Portal (PANTHER Overrepresentation Test accessed on 13.04.2017; PANTHER version 12.0 accessed on 10.07.2017, http://geneontology.org)^56^. The adjustment for multiple comparisons was made using the FDR test. The networks of intergene interaction were inferred using GeneMANIA (version 3.5.0, accessed on 13 March 2017, http://genemania.org) and the automatic weighting for the network^57^.

## Supplementary Information

Supplementary Table 1.

Supplementary Table 2.

Supplementary Table 3.

Supplementary Table 4.

Supplementary Table 5.

Supplementary Table 6.

Supplementary Table 7.

Supplementary Table 8.
