# Supplementary material for: Polymorphisms of the matrix metalloproteinase genes are associated with essential hypertension in a Caucasian population of Central Russia
Source: Sci Rep. 2021 Mar 4;11:5224. doi: 10.1038/s41598-021-84645-4 (PMC7933364; doi:10.1038/s41598-021-84645-4)
Supplement: Supplementary file 1 — Supplementary Table 1. [file 41598_2021_84645_MOESM1_ESM.doc]

Supplementary table 1. Genotype combinations associated with the risk of EH*

| N | Genotype combinations | Number of cases | Number of controls | Beta | *P* | Risk, High/Low |
| --- | --- | --- | --- | --- | --- | --- |
| 1 | rs11225395 TT × rs1320632 AA | 147 | 99 | -0.453 | 0.014 | L |
| 2 | rs1320632 AG × rs11568818 СС | 10 | 12 | -1.410 | 0.027 | L |
| 3 | rs1320632 AA × rs11568818 CC × rs3025058 6A/6A | 36 | 25 | -0.643 | 0.030 | L |
|  | rs1320632 AG × rs11568818 CT × rs3025058 5A/5A | 13 | 16 | -0.987 | 0.021 | L |
| 4 | rs11225395 CC × rs1320632 AA × rs11568818 TT | 82 | 28 | 0.497 | 0.043 | H |
|  | rs11225395 CT × rs1320632 AG × rs11568818 CC | 42 | 34 | -0.827 | 0.002 | L |
| 5 | rs17577 AG × rs1320632 AG × rs11568818 CT | 20 | 22 | -0.975 | 0.006 | L |
|  | rs17577 AG × rs1320632 AA × rs11568818 CC | 33 | 31 | -0.831 | 0.004 | L |
| 6 | rs1320632 AG × rs11568818 CT × rs17997501 1G/1G | 18 | 18 | -1.022 | 0.010 | L |
|  | rs1320632 AG × rs11568818 CC × rs179975011G/1G | 6 | 9 | -1.341 | 0.044 | L |
|  | rs1320632 AA × rs11568818 CC × rs179975011G/2G | 48 | 38 | -0.579 | 0.021 | L |
| 7 | rs11225395 CC × rs1320632 AA × rs3025058 5A/6A | 153 | 56 | 0.412 | 0.024 | H |
|  | rs11225395 CC × rs1320632 AA × rs3025058 5A/5A | 30 | 22 | -0.690 | 0.036 | L |
|  | rs11225395 CT × rs1320632 AG × rs3025058 5A/5A | 16 | 18 | -0.843 | 0.035 | L |
| 8 | rs652438 AA × rs11225395 CT × rs1320632 AG × rs11568818 CT | 38 | 32 | -0.847 | 0.003 | L |
|  | rs652438 AA × rs11225395 CC × rs1320632 AA × rs11568818 CC | 47 | 31 | -0.615 | 0.022 | L |
|  | rs652438 AA × rs11225395 TT × rs1320632 AA × rs11568818 CC | 17 | 21 | -0.770 | 0.035 | L |

Supplementary table 1 (continued)*

| N | Genotype combinations | Number of cases | Number of controls | Beta | *P* | Risk, High/Low |
| --- | --- | --- | --- | --- | --- | --- |
| 9 | rs652438 AA × rs1320632 AA × rs11568818 CC × rs3025058 5A/5A | 31 | 22 | -0.716 | 0.024 | L |
|  | rs652438 AA × rs1320632 AG × rs11568818 CT × rs3025058 5A/5A | 13 | 16 | -0.987 | 0.021 | L |
|  | rs652438 AA × rs1320632 AA × rs11568818 CC × rs3025058 5A/5A | 21 | 19 | -0.759 | 0.037 | L |
| 10 | rs1320632 AA × rs11568818 CC × rs3025058 6A/6A × rs243865 CC | 17 | 15 | -0.998 | 0.012 | L |
|  | rs1320632 AG × rs11568818 CT × rs3025058 5A/5A × rs243865CC | 7 | 13 | -1.348 | 0.011 | L |
|  | rs1320632 AA × rs11568818 CC × rs3025058 5A/5A × rs243865 CT | 6 | 6 | -1.481 | 0.024 | L |
|  | rs1320632 AA × rs11568818 CT × rs3025058 5A/6A × rs243865 TT | 11 | 2 | 1.701 | 0.043 | H |
| 11 | rs11225395 CT × rs1320632 AG × rs11568818 CT × rs243865CC | 23 | 21 | -1.047 | 0.004 | L |
|  | rs11225395 TT × rs1320632 AA × rs11568818 CC × rs243865 CC | 8 | 17 | -0.960 | 0.033 | L |
|  | rs11225395 CC × rs1320632 AA × rs11568818 CC × rs243865 CT | 18 | 15 | -0.781 | 0.044 | L |
| 12 | rs17577 AG × rs1320632 AG × rs11568818 CT × rs1799750 1G/1G | 7 | 13 | -1.726 | 0.002 | L |
|  | rs17577 AG × rs1320632 AA × rs11568818 CC × rs1799750 1G/2G | 14 | 15 | -1.045 | 0.017 | L |

***** Genotype combinations are derived from the interaction models obtained by the MB-MDR method and described in tables 4-5
