# Supplementary material for: Polymorphisms of the matrix metalloproteinase genes are associated with essential hypertension in a Caucasian population of Central Russia
Source: Sci Rep. 2021 Mar 4;11:5224. doi: 10.1038/s41598-021-84645-4 (PMC7933364; doi:10.1038/s41598-021-84645-4)
Supplement: Supplementary file 3 — Supplementary Table 3. [file 41598_2021_84645_MOESM3_ESM.doc]

Supplementary table 3. Regulatory effects of the SNPs selected for the study and their effect on gene expression

| **SNPs** | **Regulatory effects** | | | | | | | | | | | | **Expression QTLs** | |
| --- | --- | --- | --- | --- | --- | --- | --- | --- | --- | --- | --- | --- | --- | --- |
|  | **HaploReg^1^** | | | | | | **SNPinfo^2^** | | | | | |  |  |
|  | **SiPhy cons** | **Prom histone marks** | **Enh histone marks** | **DNAse** | **Proteins bound** | **Motifs changed** | **TFBS** | **Splicing (ESE/ESS)** | **microRNA (Sanger)** | **ns SNP** | **Regulatory potential** | **Conservation** | **Haplo Reg** | **GTEx portal** |
| rs1799750  *МMР1* |  |  | 5 tiss | 5 tiss | CFOS, GATA2 | 21 altered motifs | – | – | – | – | 0,01 | 0,002 | 8 hits |  |
| rs243865  *МMР2* |  |  | 11 tiss | ESDR,  IPSC,MUS |  | Myf | yes | – | – | – | 0,02 | 0,003 | 1 hit |  |
| rs3025058  *МMР3* |  |  | GI, SKIN | THYM |  | CIZ,Gfi1b | – | – | – | – | 0,0 | 0,001 |  |  |
| rs11568818  *МMР7* | yes | 4 tiss | 7 tiss | 15 tiss | TBP, CFOS,CJUN | 4 altered motifs | yes | – | – | – | 0,08 | 0,075 | 4 hits | 5 tiss |
| rs1320632  *МMР8* |  |  | 6 tiss | 10 tiss |  | 7 altered motifs | yes | – | – | – | 0,53 | 0,000 | 3 hits | 4 tiss |
| rs11225395  *МMР8* |  |  | 6 tiss | SKIN |  |  | yes | – | – | – | 0,20 | 0,000 | 3 hits | 7 tiss |
| rs17577  *МMР9* | yes | 8 tiss | 16 tiss | 22 tiss | 4 bound proteins |  | – | yes | yes | yes | NA | 0,996 | 6 hits | 13 tiss |
| rs652438  *МMР12* |  |  | GI |  |  |  | – | yes | – | yes | 0,15 | 0,000 |  |  |

Note:

^1^ Enh – Enhancer, Prom – Promoter, DNAse – DNAse hypersensitive regions; motifs – Regulatory motifs changed; tiss – tissues;

^2^ TFBS, Transcription Factor Binding Sites; ESE, Exonic Splicing Enhancer; ESS, Exonic Splicing Silencer; Polyphen, predicted damaging nsSNPs; RegPotential, Regulatory Potential Score TFBS – transcription factor binding site; ESE (Eхonic Splicing Enhancer), ESS – Exonic Splicing Silencer; nsSNP – non-synonymous SNPs; сonservation – conservative sequence
