# Supplementary material for: Polymorphisms of the matrix metalloproteinase genes are associated with essential hypertension in a Caucasian population of Central Russia
Source: Sci Rep. 2021 Mar 4;11:5224. doi: 10.1038/s41598-021-84645-4 (PMC7933364; doi:10.1038/s41598-021-84645-4)
Supplement: Supplementary file 5 — Supplementary Table 5. [file 41598_2021_84645_MOESM5_ESM.docx]

Supplementary table 5. Effect of four EH-associated SNPs on gene expression level (*cis*-eQTL) in various tissues *

| N | SNP | Gene Symbol | Ref | Alt | Effect Size (β) | p-value | N | Tissue |
| --- | --- | --- | --- | --- | --- | --- | --- | --- |
| 1. | rs11568818 | *ММP7* | T | C | -0.351 | 8.1e-14 | 383 | Lung |
|  |  | *ММP7* | T | C | -0.312 | 5.1e-11 | 220 | Pancreas |
|  |  | *ММP7* | T | C | -0.230 | 3.9e-8 | 414 | Skin - Sun Exposed (Lower leg) |
|  |  | *ММP7* | T | C | -0.318 | 0.0000019 | 237 | Stomach |
|  |  | *ММP7* | T | C | -0.359 | 0.0000035 | 132 | Prostate |
| 2. | rs1320632 | *ММР27* | C | T | -0.339 | 3.0e-9 | 385 | Adipose - Subcutaneous |
|  |  | *ММР27* | C | T | -0.467 | 1.2e-8 | 491 | Muscle - Skeletal |
|  |  | *ММР27* | C | T | -0.544 | 3.6e-8 | 361 | Nerve - Tibial |
|  |  | *ММР27* | C | T | -0.263 | 6.7e-7 | 414 | Skin - Sun Exposed (Lower leg) |
| 3. | rs11225395 | *MMP27* | A | G | -0.190 | 2.4e-10 | 414 | Skin - Sun Exposed (Lower leg) |
|  |  | *MMP27* | A | G | 0.277 | 4.8e-9 | 369 | Whole Blood |
|  |  | *MMP27* | A | G | -0.207 | 1.1e-8 | 385 | Adipose - Subcutaneous |
|  |  | *MMP27* | A | G | -0.208 | 0.0000022 | 335 | Skin - Not Sun Exposed (Suprapubic) |
|  |  | *MMP27* | A | G | -0.218 | 0.0000092 | 491 | Muscle - Skeletal |
|  |  | *RP11-817J15.3* | A | G | -0.413 | 0.000021 | 122 | Small Intestine - Terminal Ileum |
|  |  | *RP11-817J15.3* | A | G | 0.187 | 0.000038 | 369 | Whole Blood |
| 4. | rs17577 | *SLC12A5* | G | A | 0.661 | 8.9e-22 | 383 | Lung |
|  |  | *SLC12A5* | G | A | 0.818 | 8.6e-17 | 313 | Adipose - Visceral (Omentum) |
|  |  | *SLC12A5* | G | A | 0.706 | 1.9e-15 | 385 | Adipose - Subcutaneous |
|  |  | *SLC12A5* | G | A | 0.787 | 3.2e-11 | 251 | Breast - Mammary Tissue |
|  |  | *SLC12A5* | G | A | 0.895 | 4.6e-11 | 146 | Spleen |
|  |  | *SLC12A5* | G | A | 0.657 | 3.3e-8 | 267 | Artery - Aorta |
|  |  | *SLC12A5* | G | A | 0.481 | 2.9e-7 | 399 | Thyroid |
|  |  | *SLC12A5* | G | A | 1.08 | 3.8e-7 | 101 | Uterus |
|  |  | *SLC12A5* | G | A | 0.469 | 5.3e-7 | 361 | Nerve - Tibial |
|  |  | *SLC12A5* | G | A | 0.438 | 0.0000011 | 414 | Skin - Sun Exposed (Lower leg) |
|  |  | *SNX21* | G | A | 0.192 | 0.0000065 | 491 | Muscle - Skeletal |
|  |  | *SLC12A5* | G | A | 0.659 | 0.000014 | 152 | Artery - Coronary |
|  |  | *SLC12A5* | G | A | 0.441 | 0.000031 | 335 | Skin - Not Sun Exposed (Suprapubic) |

Note: * - Genotype-Tissue Expression (GTEx) project data (GTEx Consortium, 2015) (<http://www.gtexportal.org/>) (р <8.0е-5, FDR≤0.05)
