# Supplementary material for: Polymorphisms of the matrix metalloproteinase genes are associated with essential hypertension in a Caucasian population of Central Russia
Source: Sci Rep. 2021 Mar 4;11:5224. doi: 10.1038/s41598-021-84645-4 (PMC7933364; doi:10.1038/s41598-021-84645-4)
Supplement: Supplementary file 7 — Supplementary Table 7. [file 41598_2021_84645_MOESM7_ESM.doc]

Supplementary table 7. Gene set enrichment analysis of biological pathways with EH-associated genes (Gene Ontology (GO) Portal tools - PANTHER Overrepresentation Test accessed on 13.04.2017; PANTHER version 12.0 accessed on 10.07.2017, [http://geneontology.org](http://geneontology.org/)).

| Database | Gene set | Original # genes in gene set | # genes in gene set analyzed by Gene Ontology | Fold Enrichment | P_FDR_ |
| --- | --- | --- | --- | --- | --- |
| GO biological process complete | collagen catabolic process | 43 | 7 | > 100 | 4.68E-15 |
|  | extracellular matrix disassembly | 66 | 7 | > 100 | 4.79E-14 |
|  | regulation of neuroinflammatory response | 33 | 3 | > 100 | 2.63E-04 |
|  | response to amyloid-beta | 48 | 4 | > 100 | 1.03E-07 |
|  | endodermal cell differentiation | 41 | 3 | > 100 | 4.42E-04 |
|  | positive regulation of vascular associated smooth muscle cell proliferation | 35 | 2 | > 100 | 6.39E-05 |
|  | cellular response to reactive oxygen species | 133 | 3 | 67.19 | 9.31E-06 |
|  | female pregnancy | 189 | 3 | 47.28 | 2.62E-05 |
|  | cytokine-mediated signaling pathway | 696 | 4 | 17.12 | 4.06E-05 |
|  | proteolysis | 1287 | 7 | 16.20 | 3.48E-09 |
| GO molecular function complete | metalloendopeptidase activity | 109 | 7 | > 100 | 1.37E-16 |
|  | serine-type endopeptidase activity | 172 | 6 | > 100 | 3.89E-09 |
|  | zinc ion binding | 838 | 7 | 24.88 | 1.75E-10 |
| Reactome pathways | Activation of Matrix Metalloproteinases | 33 | 6 | > 100 | 2.28E-13 |
|  | Collagen degradation | 140 | 7 | > 100 | 8.92E-15 |
|  | Extra-nuclear estrogen signaling | 73 | 4 | > 100 | 2.72E-06 |
|  | Assembly of collagen fibrils and other multimeric structures | 60 | 3 | > 100 | 2.31E-04 |
|  | EPH-ephrin mediated repulsion of cells | 50 | 2 | > 100 | 2.23E-02 |
|  | Interleukin-4 and Interleukin-13 signaling | 111 | 4 | > 100 | 1.15E-05 |
