# Supplementary material for: Polymorphisms of the matrix metalloproteinase genes are associated with essential hypertension in a Caucasian population of Central Russia
Source: Sci Rep. 2021 Mar 4;11:5224. doi: 10.1038/s41598-021-84645-4 (PMC7933364; doi:10.1038/s41598-021-84645-4)
Supplement: Supplementary file 8 — Supplementary Table 8. [file 41598_2021_84645_MOESM8_ESM.doc]

**Supplementary table 8. Biological functions of polymorphisms of genes of matrix metalloproteinases and their association with EН**

| Gene name  (Gene ID) | Protein function | Polymorphism, suggested effect/function (reference) |
| --- | --- | --- |
| *MMP1*,  matrix metalloproteinase 1, collagenase-1 | MMP1 is an important enzyme capable of hydrolyzing interstitial fibrillar collagen fibers. Substrates can be collagen of types I, II, III, VII, X, XI, gelatin, aggrecan, casein, entactin, fibronectin, laminin, vitronectin, serpins, α2-macroglobulin, fibrinogen, fibroblast growth factor (FGF), interleukin (IL1β) . This protein is secreted by macrophages, osteoblasts, endotheliocytes, fibroblasts and is involved in tissue remodeling, regeneration processes, and embryo development. | rs1799750 (-1607 1G/2G). Genotype 1G/1G is characterized by low transcriptional activity (compared to genotypes 1G/2G and 2G/2G), which leads to the accumulation of extracellular matrix and pathological stenosis of arteries. Carrier genotype 2G/2G, on the contrary, leads to an increase in transcriptional activity of the gene, which potentially leads to an increase in the rate of collagen breakdown (Lin C.C., Yang W.C., 2013). In the Mexican cohort, there were no significant differences in the frequencies of alleles and genotypes between the group of patients with essential hypertension and the control group (p<0.05). In a study by Velho F.M. et al. (2011) showed that the genetic variant -1607 2G is involved in the development of myocardial infarction in patients with EH in the Brazilian population (OR=0.47, 95% CI=0.27-0.82, p=0.008). The -1607 2G allele increases the risk of atherosclerotic vascular damage in the Serbian population (OR=3.49, 95% CI=1.67-7.30, p=0.0009) (Djuric T. et al., 2014) However, this polymorphism is not associated with atherosclerosis in the Australian population (Morris D.R. et al., 2014). |
| *MMP2*,  matrix metalloproteinase 2 | MMP2 belongs to the subfamily of gelatinases and is responsible for the hydrolysis of type IV collagen in the basement membranes. MMP-2 is synthesized by endotheliocytes, smooth muscle cells of the vessel wall, monocytes, keratinocytes, fibroblast cells and is involved in the most important biological processes - angiogenesis, maturation of blood vessels, osteogenesis, breast involution, tissue regeneration and remodeling, ovulation and implantation. | **rs243865** (-1306 С/Т). Genotype CC is associated with a decrease in the left ventricular mass index in patients with EН (р=0.0365) n the Brazilian population (Lacchini R. et al., 2012). However, these data are not consistent with the results of a study by the Metzger I.F. et al. (2012), which in the course of a meta-analysis revealed no associations between rs243865 polymorphism and the development of cardiovascular pathology. Multivariate logistic regression analysis did not reveal an association between rs243865 polymorphism and the development of ischemic stroke in patients with essential hypertension in the Chinese population (Hao Y., 2015 The rs243865 polymorphism is associated with a high risk of developing intracranial aneurysm due to EH in the Japanese population (p=0.0009) of the development of cardiovascular pathology in the Japanese population (Low S.K. et al. 2011).  rs243866 (-1575 A/G). The -1575 A allele is associated with the development of EH and higher levels of neopterin, total cholesterol, triglycerides and low HDL levels (OR=1.78, 95% СI=1.23-2.06, р=0.029) in the Iranian population (Bahrehmand F. et al., 2012).  rs2285053 (-735 С/T). The -735 C allele is associated with the occurrence of unstable atherosclerotic plaques in individuals of the Chinese Hanzi population (ОR=1.438, 95% СI=1.089-1.519, р=0.004) (Wang F. et al., 2011). |
| *MMP3*,  matrix metalloproteinase 3, stromelysin 1 | MMP3 is characterized by low substrate specificity and is responsible for the hydrolytic cleavage of fibronectin. laminin, collagen III, IV, IX and X, gelatins I, III, IV and V, cartilaginous proteoglycans. Stromelysin-1 is synthesized by most cells of connective tissue and is involved in tissue regeneration after damage, atherogenesis, the onset and growth of tumors. | rs35068180 (-1171 5А/6А). In 5A/6A heterozygotes, the level of gene expression is optimal for remodeling and maintaining vascular elasticity, which reduces the risk of hypertension and other cardiovascular diseases in the Australian population (Medley T. L. et al., 2013).  **rs3025058** (-1612 5A/6A). Multivariate logistic regression analysis showed a lack of correlation between polymorphism and the occurrence of essential hypertension in the Chinese population (Hao Y. et al., 2015). Allele -1612 6A *MMP3* correlates with the progression of narrowing of the lumen and the development of acute myocardial infarction in patients with EH in the Polish population (OR=1.568, 95% СI=1.201-2.048, р=0.008) (Sakowicz A. et al. 2015). Genotype 5A/5A is associated with a higher (5-fold) risk of complications after acute myocardial infarction in US residents (El-Aziz T.A. et al., 2016). The association of rs3025058 polymorphism with the development of myocardial infarction in patients with arterial hypertension in the Mexican population has not been correlated (Rodriguez-Perez J.M. et al., 2016). The -1612 6A polymorphic marker is associated with increased blood pressure and aortic rupture in Australia (OR=1.48; 95% СI=1.23-1,78, р=3.95×10^-5^) (Morris D.R. et al., 2014). |
| *MMP7*,  matrix metalloproteinase 7, matrilysin-1 | MMP7 breaks down collagen IV, aggrecan, fibronectin, gelatins I, III, IV, V, laminin, casein, entactin, elastin, versican, as well as osteopontin. MMP7 secretion is carried out by normal and pathologically altered epithelial cells, endotheliocytes, chondrocytes, as well as cells of exocrine glands, endometrium, and tumors. Matrilizin-1 is involved in embryogenesis, migration, proliferation and apoptosis of epithelial cells, postpartum uterine involution, bone remodeling. | rs11568818 (-181 A/G), rs11568819 (-153 C/T). Genotype GG (rs11568818) and genotype TT (rs11568819) are associated with the occurrence of arterial hypertension and atherosclerotic vascular lesions in the Swiss population (Jormsjö S. et al., 2011). The -181 A/G *MMP7* polymorphism is not associated with the occurrence of arterial hypertension in the Indian population (Mishra A. et al., 2012) and is not associated with a genetic predisposition to myocardial infarction due to EH in the Mexican population (Pérez-Hernández N. et al., 2012). |
| *MMP8*,  matrix metalloproteinase 8, neutrophil collagenase | MMP8 is an endopeptidase of the collagenase subfamily and is responsible for the hydrolysis of a wide range of fibrillar (types I, II, III, V and XI) and non-fibrillar (types IX, XII and XIV) collagens. MMP-8 secretion is carried out by cells of the neutrophil line, endotheliocytes, pericytes, fibroblasts, chondrocytes. MMP8 activates a number of chemokines and growth factors, thus affecting cell proliferation, cell differentiation, apoptosis, angiogenesis, oncogenesis and metastasis. | rs11225395 (799 С/Т). The promoter region carrying the -799 T mutant allele of the *MMP8* gene is characterized by increased activity compared to the promoter containing the wild-type -799 C allele. The 799 T allele is associated with the occurrence of an aortic aneurysm in the Chinese population (Wang X. et al., 2013), with the development of cardiovascular disease in the Iranian population (Hoseini S.M. et al., 2015).  rs1320632 (-381A/G). Polymorphism is a risk factor for carotid arteriosclerosis in the Serbian population. The frequency of the -381G allele is significantly higher in the group of patients with atherosclerosis than in the control group (OR=1.7, 95% CI=1.1-2.9, p=0.001) (Djuric T. et al., 2014). |
| *MMP9*,  matrix metalloproteinase 9, gelatinase B | MMP9 is responsible for the proteolysis of denatured type I collagen, as well as gelatins, fibronectin, collagens (type V, VII, X, XIV), interleukins, fibrinogen, and entactin. Endopeptidase inactivates the inhibitors of MMP - α2-macroglobulin and α1-proteinase inhibitor and acts as an inducer for cytokines and growth factors. Gelatinase B is involved in processes such as inflammation, tissue remodeling and regeneration, embryogenesis. By destroying the extracellular matrix of blood vessels, MMP9 promotes the release of VEGF and activates angiogenesis. | rs17577 (688 G/A) Genotype AA is associated with a high risk of developing cardiovascular disease in individuals with obesity in the Brazilian population (Luizon M.R. et al., 2016). There are no associations with the development of ischemic stroke in the Chinese population (Hao Y. et al., 2015).  rs3918242 (-1562 С/Т), rs17576 (-836 А/G). Carriers of mutant alleles (-1562 T and -836 G, respectively) show a higher activity of the promoter region of the gene due to the binding of the transcription repressor, which leads to excessive accumulation of the enzyme and excessive degradation of the extracellular matrix in the vascular wall (Metzger I.F. et al., 2012). The rs3918242 polymorphism is associated with essential hypertension in the Chinese population (OR=1.36, 95% CI=1.17-1.59, p=0.0001) (Yang W. et al., 2015) and in the Mexican population (OR=2.88, 95% CI=1.68-3.92, p<0.01) (Rodriguez-Perez JM et al. 2016). The T allele rs3918242 correlates with a high risk of myocardial infarction in the Polish population (OR=1.14, 95% CI=1.02-1.27; p=0.02) (Sakowicz A. et al. 2015). The rs17576 *MMP9* polymorphism is associated with the development of left ventricular dysfunction in the Indian population (OR=3.82, 95% CI=2.11-4.8 p=0.009) (Mishra A. et al., 2012). |
| *MMP12*,  matrix metalloproteinase 12, macrophage metalloelastase | MMP12 has potent elastolytic activity and broad substrate specificity similar to stromelysins. Hydrolyzes elastin, collagen IV, fibronectin, gelatin, vitronectin, proteoglycan, plasminogen, laminin, fibrinogen, a2-macroglobulin. Increased macrophage elastase values are observed in smoker's alveolar macrophages, as well as inflammation, embryogenesis, atherogenesis, tumor growth and invasion, angiogenesis, and tissue regeneration. | rs2276109 (-82 А/G) The -82 G polymorphic variant is associated with a high risk of developing cardiovascular disease in the US population (OR=1.395, 95% CI=1.049-1.956, p=0.02) (Tanner R.M. et al., 2011). The -82 G allele is associated with susceptibility to aneurysm in German residents (OR=1.26, 95% CI 1.07-1.89, p=0.011) (Arning A. et al., 2016), however, there are no associations rs2276109 with aneurysm in the Australian population (Morris DR et al., 2014). Allele G rs652438 is associated with the development of ischemic stroke in Europeans (OR=2.54 95% CI=1.34-4.80 in the dominant model, p=0.004) and Africa (OR=5.77, 95% CI=3.42-9.71 in the dominant model, p=0.0001) (Zhang G. et al., 2018), coronary heart disease in the American population (OR=2.47, p=0.01) (Lynch AI et al., 2012), as well as aortic aneurysms in the Italian population (OR=2.8, 95% CI=1.3-6.0, p=0.008) (Fiotti N. et al., 2018). |
